# Effect of Different Filler Contents and Printing Directions on the Mechanical Properties for Photopolymer Resins

**DOI:** 10.3390/ijms23042296

**Published:** 2022-02-18

**Authors:** Tamaki Hada, Manabu Kanazawa, Nanako Miyamoto, Hengyi Liu, Maiko Iwaki, Yuriko Komagamine, Shunsuke Minakuchi

**Affiliations:** 1 Gerodontology and Oral Rehabilitation, Graduate School of Medical and Dental Sciences, Tokyo Medical and Dental University, 1-5-45 Yushima, Bunkyo-ku, Tokyo 113-8549, Japan; t.hada.gerd@tmd.ac.jp (T.H.); h.liu.gerd@tmd.ac.jp (H.L.); y.komagamine.gerd@tmd.ac.jp (Y.K.); s.minakuchi.gerd@tmd.ac.jp (S.M.); 2 Digital Dentistry, Graduate School of Medical and Dental Sciences, Tokyo Medical and Dental University, 1-5-45 Yushima, Bunkyo-ku, Tokyo 113-8549, Japan; 3 Faculty of Dentistry, Tokyo Medical and Dental University, 1-5-45 Yushima, Bunkyo-ku, Tokyo 113-8549, Japan; 180512ds@tmd.ac.jp; 4 Oral Prosthetic Engineering, Graduate School of Medical and Dental Sciences, Tokyo Medical and Dental University, 1-5-45 Yushima, Bunkyo-ku, Tokyo 113-8549, Japan; m.iwaki.gerd@tmd.ac.jp

**Keywords:** three-dimensional (3D) printing, polymethyl methacrylate (PMMA), zirconia filler, composite, printing direction, mechanical properties

## Abstract

Photopolymer resins are widely used in the production of dental prostheses, but their mechanical properties require improvement. We evaluated the effects of different zirconia filler contents and printing directions on the mechanical properties of photopolymer resin. Three-dimensional (3D) printing was used to fabricate specimens using composite photopolymers with 0 (control), 3, 5, and 10 wt.% zirconia filler. Two printing directions for fabricating rectangular specimens (25 mm × 2 mm × 2 mm) and disk-shaped specimens (φ10 mm × 2 mm) were used, 0° and 90°. Three-point bending tests were performed to determine the flexural strengths and moduli of the specimens. The Vickers hardness test was performed to determine the hardness of the specimens. Tukey’s multiple comparison tests were performed on the average values of the flexural strengths, elastic moduli, and Vickers hardness after one-way ANOVA (α = 0.05). The flexural strengths and elastic moduli at 0° from high to low were in the order of 0, 3, 10, and 5 wt.%, and those at 90° were in the order of 3, 0, 10, and 5 wt.% (*p* < 0.05). For 5 and 10 wt.%, no significant differences were observed in mechanical properties at 0° and 90° (*p* < 0.05). The Vickers hardness values at 0° and 90° from low to high were in the order of 0, 3, 5, and 10 wt.% (*p* < 0.05). Within the limits of this study, the optimal zirconia filler content in the photopolymer resin for 3D printing was 0 wt.% at 0° and 3 wt.% at 90°.

## 1. Introduction

In recent years, computer-aided design and manufacturing (CAD/CAM) technologies have been used in some medical and dental treatments, and the digitization of prosthesis production has been progressing [1,2,3,4]. Prosthesis production by printed molding is on the rise because it is inexpensive and time-efficient and enables ready manufacturing of three-dimensional (3D)-printed prostheses. Therefore, several dental laboratories and clinics have introduced stereolithography (SLA) and digital light processing (DLP) 3D printers using photopolymer resins.

Polymethyl methacrylate (PMMA) has been used as a raw material in several photopolymer resins for 3D printing. PMMA is the most commonly used material in dental prostheses owing to its excellent dimensional stability in the oral environment, low odor, low irritation, low cost, light weight, favorable esthetics, and ease of fabrication and repair. However, several challenges, such as fracture due to water absorption, decrease in flexural and impact strengths, porosity, and polymerization shrinkage, limit the use of PMMA [5,6,7]. Therefore, it is necessary to enhance the mechanical properties of PMMA denture base materials. Contemporary studies have focused on reinforcement methods, such as the insertion of reinforcing wires and frameworks and the addition of fibers and micro- or nanosized fillers [8,9].

One study [10] proposed the insertion of metal wires and plates for the reinforcement of conventional PMMA denture base materials and reported improved strength of the modified PMMA in the lateral direction. The use of carbon, aramid, glass, and polyethylene fibers with ultrahigh elastic moduli as reinforcing materials has also been investigated. Zirconia has been commercialized owing to its excellent biocompatibility, high bending strength (900–1200 MPa), high hardness (1200 HV), and fracture toughness (9–10 MPa m^1/2^). Zidan et al. [11] studied the mechanical properties (flexural strength, fracture toughness, impact strength, and hardness) and fracture behavior of high-impact heat-cured PMMA with varying amounts of an yttria-stabilized zirconia filler. They reported that PMMA achieved the optimum mechanical properties for use as prosthesis beds upon the addition of 3–5 wt.% zirconia filler. Thus, this method can be used to overcome the challenges of unfavorable mechanical properties and brittleness of PMMA.

Typically, for a specific range of filler content, a higher filler content implies superior mechanical properties and wear resistance of the composite resin. Therefore, several studies have investigated the addition of micro- and nanosized fillers to photopolymer resins for 3D printing to improve their mechanical properties and overcome their limitations [12,13] such as shrinkage, brittleness, and unfavorable mechanical properties during photocuring. Mubarak et al. [14] added <1 wt.% of Ag-TiO_2_ nanofiller to a PMMA-based photopolymer resin, and it improved the mechanical properties, thermal conductivity, and thermal stability of the resin. Mohan et al. [15] reported improved mechanical properties of a PMMA-based photopolymer resin upon the addition of <3 wt.% of cellulose nanofibrils (CNFs); in particular, the tensile strength improved by 37%. Chen et al. [16] prepared PMMA-based photopolymer resins using various concentrations of polyetheretherketone (PEEK) microfillers and TiO_2_ nanofillers. They evaluated the mechanical properties, filler distribution, and antibacterial activity, among other characteristics of the prepared resins; 1 wt.% of TiO_2_ and 1 wt.% of PEEK effectively enhanced the mechanical and antibacterial properties of the PMMA composite resin. However, the addition of zirconia [17], a bioceramic material that is widely used in various dental restorations, implant screws and abutments, and orthodontic brackets, to photopolymer resin as a filler has not been reported.

Furthermore, a few studies [18,19,20,21] have reported that the mechanical properties of products manufactured using SLA and DLP 3D printers are affected by the printing directions. Shim et al. [22] reported that, compared with that in the printing direction parallel to the loading direction (90°), the flexural strength was higher when the printing direction of the specimens was perpendicular to the loading direction (0°). However, these reports have focused on pure photopolymer resins without fillers; therefore, it is necessary to examine the effects of the printing direction when fillers are added to the resin.

This study aimed to evaluate the effects of different zirconia filler contents and printing directions (0° and 90°) on the mechanical properties for 3D printing of the photopolymer resin. The null hypothesis is that there is no difference in mechanical properties due to the difference in the amount of zirconia filler added in different printing directions (0° and 90°).

## 2. Results

### 2.1. Particle Size and Distribution of Zirconia Filler

**Figure 1:**
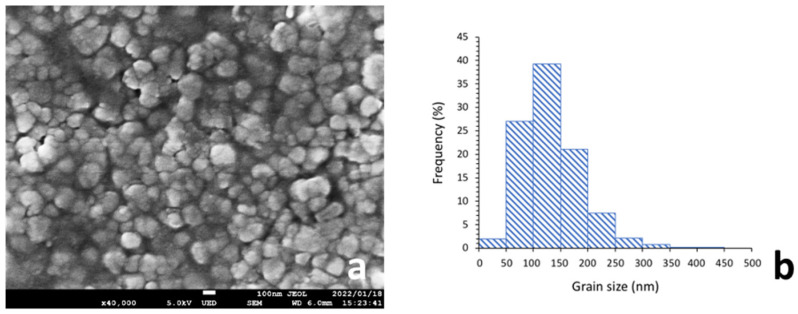
Particle size (40,000× microscopic images) and distribution of zirconia filler at 10 wt.%: (**a**) Particle size determined by the linear intercept method (average 206 µm) and (**b**) Minimum 600 grains measured, with the highest size distribution between 100 nm and 150 nm.

**Figure 2:**
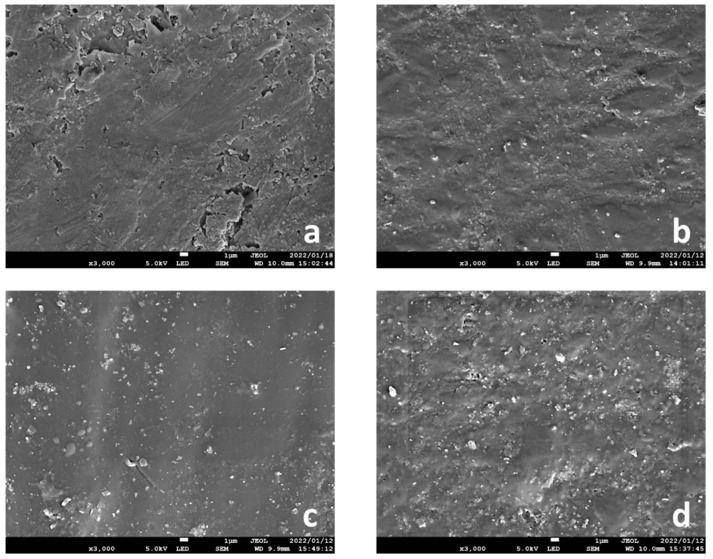
Distribution of zirconia filler under each condition (3000× microscope image). The zirconia filler was uniformly distributed in the photopolymer resin under each condition: (**a**) 0 wt.% (control), (**b**) 3 wt.%, (**c**) 5 wt.%, and (**d**) 10 wt.%. All images were captured when the printing direction was 0°.

The average particle size was 206 µm, and the highest particle size distribution was between 100 nm and 150 nm (Figure 1). Microscopic images at 3000× showed that the zirconia filler in each condition was uniformly dispersed in the photopolymer resin (Figure 2).

### 2.2. Three-Point Bending Test

Table 1 and Table 2 present the results of the flexural strength and modulus tests. The flexural strength and flexural modulus varied significantly with the zirconia content, except between 5 and 10 wt.% (*p* < 0.05). When the printing direction was 0°, the composite resins with zirconia contents of 0 (control), 3, 10, and 5 wt.% (*p* < 0.05) were arranged in the decreasing order of their flexural strengths and moduli. The highest flexural strength of 50.6 ± 0.8 MPa and flexural modulus of 0.8 ± 0.03 GPa, which met the ISO standard requirements, were observed for the resin with 0 wt.% filler content. When the printing direction was 90°, the flexural strengths and moduli of the composite resins were in the order 3, 0 (control), 10, and 5 wt.% (*p* < 0.05, decreasing order). The highest flexural strength of 51.0 ± 1.0 MPa and flexural modulus of 0.9 ± 0.04 GPa, which met the ISO standard requirements were observed for the resin with 3 wt.% zirconia content. No significant difference (*p* > 0.05) was observed between the bending strengths and elastic moduli of specimens with 5 and 10 wt.% zirconia content in both printing directions, and most specimens were fractured into two pieces at the initial stage of the three-point bending test.

### 2.3. Vickers Hardness Test

Table 3 and Table 4 present the results of the Vickers hardness tests. The Vickers hardness varied significantly with zirconia content, except between 5 and 10 wt.% when the printing direction was 90° (*p* < 0.05). When the printing direction was 0°, the composite resins with zirconia contents of 0 (control), 3, 5, and 10 wt.% (*p* < 0.05) were arranged in the increasing order of Vickers hardness. The highest Vickers hardness of 21.0 ± 0.4 VHN was observed for the resin with 10 wt.% filler content. When the printing direction was 90°, the Vickers hardness values of the composite resins were in the order of 0 (control), 3, 5, and 10 wt.% (*p* < 0.05, increasing order). The highest Vickers hardness of 21.2 ± 0.4 VHN was observed for the resin with 10 wt.% filler content. No significant difference (*p* > 0.05) was observed between the Vickers hardness values of specimens with 5 and 10 wt.% zirconia content in both printing directions.

### 2.4. SEM Analysis of Fracture Surfaces

**Figure 3:**
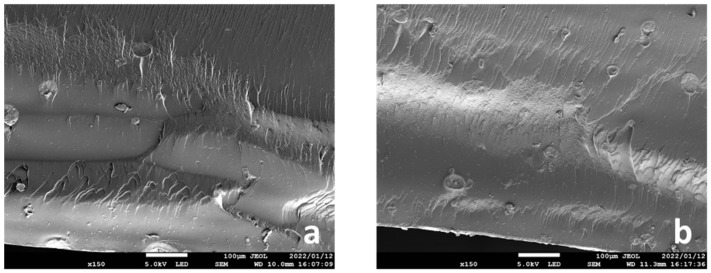
Fracture surfaces (150× microscopic image) of the specimens with (**a**) 5 wt.% and (**b**) 10 wt.% zirconia filler in the 0° printing direction observed after the three-point bending tests.

The SEM micrographs in Figure 3 show the fracture surfaces of the specimens with 5 and 10 wt.% zirconia filler added in the 0° printing direction after the three-point bending tests. The zirconia filler particles and air bubbles are embedded along the interlayer.

## 3. Discussion

In this study, different amounts of zirconia filler were added (0, 3, 10, and 5 wt.%) to the resin for printing in two different directions (0° and 90°), and their effects on the mechanical properties of the photopolymer resin for 3D printing were evaluated; the flexural strengths and moduli significantly differed under each condition. Therefore, the null hypothesis was rejected. The flexural strengths and moduli of the specimens with 5 and 10 wt.% zirconia filler were significantly lower than those of the control group. When the printing direction was set to 0°, the flexural strength and modulus of the resin containing 0 wt.% zirconia filler were significantly higher than those of the other groups, and when the printing direction was set to 90°, the flexural strength and modulus of the resin composite containing 3 wt.% zirconia filler were significantly higher than those of the other groups. The surface hardness of the specimens may be directly related to the zirconia filler content. These conditions may be used to enhance the mechanical properties of photopolymer resins used for 3D printing, thus emphasizing the significance of this study.

The flexural strengths and moduli at 5 and 10 wt.% zirconia filler in the 0° and 90° printing directions were significantly lower than those in the control group by approximately 16% and 10%, respectively. At significantly high filler concentrations, the interaction between the filler particles increases, which may decrease the fracture toughness [11]. During the three-point bending tests, most of the 5 and 10 wt.% specimens showed a tendency to fracture, indicating that the filler concentration in the photopolymer resin was extremely high. In addition, SEM images of the fractured surfaces of the specimens that fractured during the three-point bending test were captured, and many bubbles generated by the filler particles and due to filler entrapment were observed along the layers (Figure 1). A higher filler content implies better mechanical properties and wear resistance of the composite resin; however, if the amount exceeds a certain limit, the mechanical properties are negatively affected. In this study, as the zirconia filler content was increased, a load was applied during stirring of the photopolymer resin; the viscosity of the material increased with the increasing filler content. Furthermore, observation with the naked eye after lamination revealed that the surface of the specimen deteriorated with the increasing zirconia filler content. This may be attributed to the printing failure of the SLA printer at a significantly high viscosity of the photopolymer resin [23] and the variation in the accuracy of the modeled object with the viscosity of the photopolymer resin [24].

The results of this study were consistent with those of previous studies [11] reporting that the flexural strength of PMMA significantly improved with 3 wt.% yttria-stabilized zirconia filler. This indicates that the appropriate amount of zirconia filler was added to the resin in this study. This study demonstrated that the flexural characteristics obtained using 3 wt.% zirconia filler content with the 90° printing direction met the ISO10477 requirements [25]; these values are approximately 5–28% higher than the flexural strengths and moduli of the control and other test groups. The composite resin with 3 wt.% zirconia filler content and 90° printing direction exhibited mechanical properties that are clinically acceptable for producing dental prostheses. In addition, the surface of PMMA is easily damaged by mechanical cleaning due to brushing in daily life. Therefore, the hardness of the material surface is important. In this study, the Vickers hardness increased as the amount of zirconia filler increased. This trend was consistent with a previous study in which 0–5 wt.% zirconium dioxide nanoparticles were added to PMMA [26]. Therefore, the surface hardness may be directly related to the content of the zirconia filler.

Several studies [18,19,20,22,27] have demonstrated that the printing direction affects the mechanical properties of the product produced using the SLA 3D printer used in this study. Alharbi et al. [27] demonstrated that the compression strength of columnar specimens printed parallel to the loading direction were approximately 15% lower than those printed perpendicular to the loading direction. Shim et al. [22] performed three-point bending tests and reported that when the printing direction of the rectangular specimens was perpendicular (0°) to the loading direction, the bending strength was approximately 30% higher than when the printing direction was parallel (90°) to the loading direction. Similarly, in this study, for the control group, the flexural strength and the modulus tended to be higher in the 0° printing direction than in the 90° printing direction. However, the opposite tendency was observed in the test groups; the flexural strengths and moduli were higher in the 90° printing direction. Therefore, in this study, we considered the influence of the printing direction and the filler content. Gad et al. [28] reported that the surface area of the filler provided more contact points to PMMA and thus influenced the mechanical properties of the composite by improving the mechanical interlocking. Another study [22] reported that the printing direction influenced the adhesive strength between the printed layers, thereby affecting the mechanical properties; the flexural characteristics in the loading direction tended to increase along the 90° printing direction. The surface area increased due to the zirconia filler inserted between the layers, consequently improving the adhesive strength between the printed layers. However, in the 0° printing direction, the flexural strength and modulus at 0 wt.% filler content were significantly high, and the mechanical properties tended to degrade with the increasing zirconia filler content. Studies [18,19,20,22,27] have reported improved mechanical properties when the printing direction is perpendicular to the loading direction (0°). This is because, in this case, the printed layers are compressed, thus eliminating the gap between the layers, which would have led to the degradation of the mechanical properties. However, in this study, the gaps between the layers were not eliminated by the zirconia filler inserted between the layers, suggesting that the mechanical properties were degraded due to slippage or the bubbles entrapped in the filler particles.

A general-purpose 3D printer was selected for this study, and experiments were conducted using a PMMA photopolymer resin. Several studies have evaluated the physical properties of this resin; herein, a three-point bending test that provides clear interpretation of the results was conducted. Post hoc power analysis was performed using an analysis software (G*Power 3.1.9.4 software, Kiel University, Kiel, Germany). In this study, the significance level was α = 0.05, and the power 1-β was ≥0.8; therefore, the sample size in this study was appropriate. Furthermore, as zirconia, a bioceramic material, has long been widely used in various dental applications, zirconia filler can be added to photopolymer resins for 3D printing, and this may be clinically acceptable in future.

The limitation of this study is the use of an unsintered zirconia filler. The properties of a filler-reinforced resin depend on the size, shape, type, and concentration of the added particles [28]. Therefore, the mechanical properties of the resin may be further improved using a debindered sintered zirconia filler. Molecular bonds can be formed at the interface between the inorganic and organic materials by silane coupling treatment of the filler, which can increase the bond strength of the composite; thus, the results may differ depending on whether the filler is subjected to treatment. Furthermore, it was not possible to change the parameters of the 3D printer. The mechanical properties may differ due to the varying intensity or pitch of the laser beam. Therefore, to further optimize the printing conditions for enhancing the mechanical properties of the printed components, it is necessary to investigate the parameters of 3D printers as well as various photopolymer resins and specimen dimensions.

## 4. Materials and Methods

### 4.1. Material Selection

Table 5 lists the various materials used. A photopolymer resin (Clear Resin, Formlabs Inc., Somerville, MA, USA) was used as the base material for 3D printing. Highly translucent Y_2_O_3_-stabilized zirconia (Zpex4, Tosoh Corporation, Tokyo, Japan) was used as the filler to produce composite resins.

### 4.2. Determining the Optimum Mass Percentage of Zirconia Filler

To determine the optimal mass percentage of the Y_2_O_3_-stabilized zirconia filler, a preliminary survey was conducted using a photopolymer resin mixed with 0, 1.5, 3, 5, 7, and 10 wt.% zirconia filler. Based on these results and those in the related literature [11], the mass percentages of the zirconia filler used in this study were 0 (control), 3, 5, and 10 wt.%.

### 4.3. Mixing the Zirconia Filler and Photopolymer Resin

The zirconia filler and the photopolymer resin were weighed using an electronic balance (HR-100AZ, A and D, Tokyo, Japan). The filler was added to the photopolymer resin. The mixture was stirred for 10 min using a stainless-steel spatula, and the filler was uniformly dispersed in the photopolymer resin. Subsequently, the mixture was poured into the tray of a desktop SLA 3D printer (Form 2, Formlabs Inc., Somerville, MA, USA).

### 4.4. Specimen Fabrication

**Figure 4:**
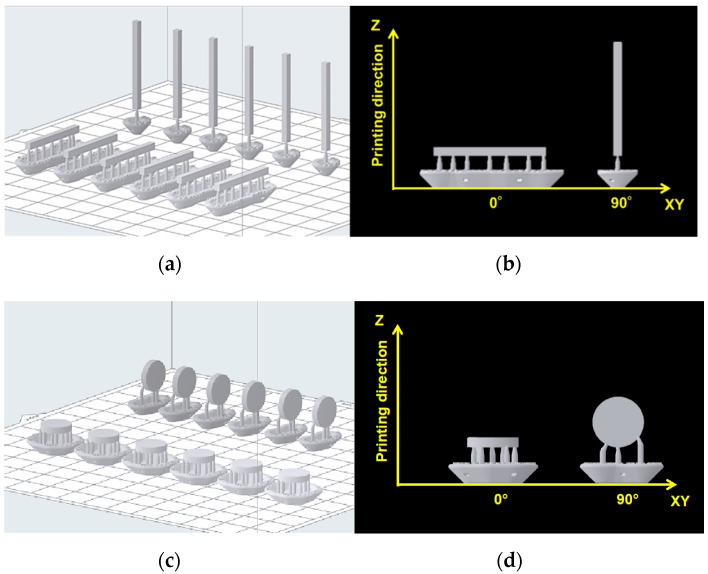
Specimens being 3D-printed in two directions: (**a**,**c**) STL data imported to the 3D printing software (n = 6); (**b**,**d**) print layer perpendicular to the loading direction (0°) and parallel to the loading direction (90°).

A 3D CAD software package (Geomagic Freeform; 3D Systems, Rock Hill, SC, USA) was used to design rectangular specimens (ISO10477, length = 25 mm, width = 2 mm, and thickness = 2 mm) and disk-shaped specimens (diameter = 10 mm and thickness = 2 mm), and the output was obtained in the standard tessellation language (STL) file format. The STL data of the specimens were imported to the 3D printing software (PreForm Software, Formlabs Inc., Somerville, MA, USA). The specimens were classified according to the printing directions (Figure 4); at 0°, the print layer was perpendicular to the loading direction, and at 90°, the print layer was parallel to the loading direction.

**Figure 5:**
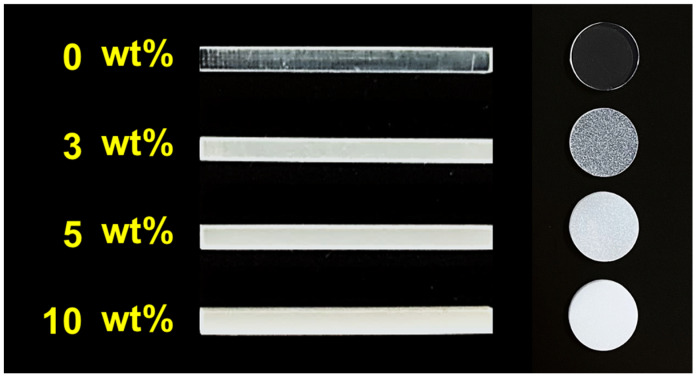
Finished specimens. Mass percentages of zirconia filler: 0 (control), 3, 5, and 10 wt.%. The specimen became opaque with increasing amounts of zirconia filler. This image was captured against a black background to make it easier to determine the color of the specimens.

A methacrylate-based photopolymer resin (layer thickness = 100 μm and infill = 100%, n = 6 for each condition) was printed using the 3D printer. After printing, the unpolymerized resin on the surface of the specimens was washed using 99.9% isopropyl alcohol (IPA) for 15 min (Form Wash, Formlabs Inc., Somerville, MA, USA). After complete drying, the specimens were post-polymerized at 40 °C for 40 min in a post-curing device (Form Cure, Formlabs Inc., Somerville, MA, USA) under light with a wavelength range of 350–500 nm. After curing, all specimens were wet-polished using a #1200 water-resistant abrasive paper and buffed (Dialap ML150P; Maruto, Tokyo, Japan) using an alumina-based abrasive (particle size = 0.3 µm) for final finishing. The dimensions of the specimens were adjusted for three-point bending tests (length × width × thickness = 25 × 2 × 2 mm^3^) according to the ISO standard [25], and Vickers hardness tests (diameter × thickness = 10 × 2 mm) and were confirmed using a digital caliper (MDC-25M; Mitutoyo, Yokohama, Japan; least count = 1 µm). The specimens were immersed in purified water at 37 ± 1 °C and stored in a constant temperature bath (DX300; Yamato, Tokyo, Japan) for 24 h (Figure 5). Therefore, it took approximately three days (~72 h) to complete curing, polishing, and mechanical property testing.

Power analysis was conducted to estimate the optimal sample size in accordance with the procedure used by Zidan et al. [11], who reported that the flexural strengths of PMMA with 3 and 10 wt.% zirconia filler were 71.5 ± 5.7 and 83.5 ± 6.2 MPa, respectively. Assuming an effect size of 1.5 and α = 0.05 and β = 0.95, the required sample size was n = 6 (G * Power 3.1.9.4 software, Kiel University, Kiel, Germany).

### 4.5. Three-Point Bending Test

**Figure 6:**
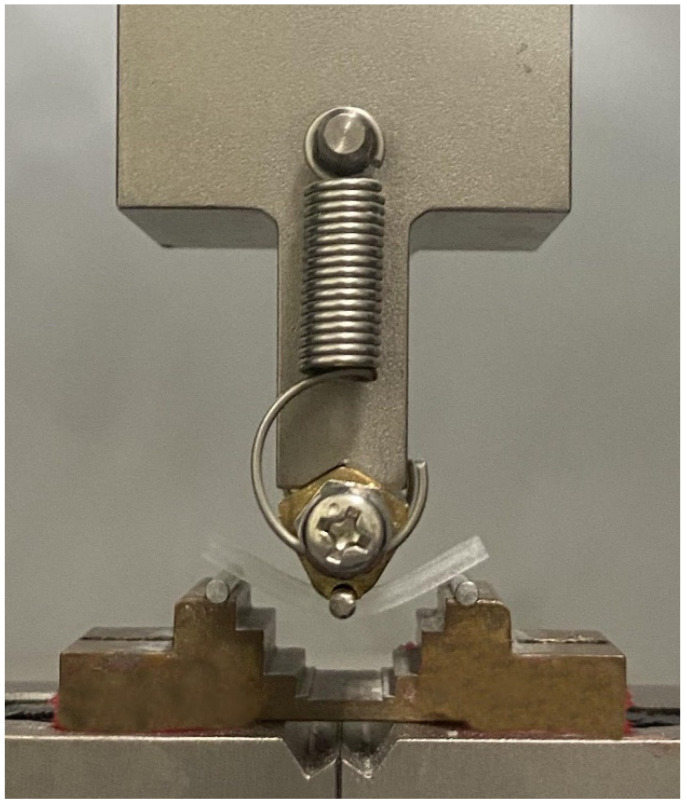
Three-point bending test setup.

The three-point bending test was performed according to the ISO10477 standard [25]. For the bending test, a rectangular specimen with a length, width, and thickness of 25, 2, and 2 mm, respectively, was used (n = 6). A precision universal testing machine (AG-Xplus; Shimadzu, Kyoto, Japan) with a span distance of 20 mm and a crosshead speed of 1 mm/min was used; load was applied using a load plunger (Figure 6) to measure the flexural strength (*FS*, MPa), and it was calculated using Equation (1):

*FS* = 3*Fl*/2*bh*^2^, (1)

where *F*, *l*, *b*, and *h* denote the maximum applied load (N), support span distance (20 mm), specimen width (mm), and specimen height (mm), respectively, prior to testing.

The flexural moduli (*FM*, GPa) of the specimens were calculated using Equation (2):

*FM* = *F*_1_*l*^3^/4*bh*^3^*d*, (2)

where *F*_1_ and *d* denote the point load (N) applied on the straight-line portion of the flexural load–deflection curve and the deflection (mm), respectively, at load *F*_1_.

### 4.6. Vickers Hardness Test

**Figure 7:**
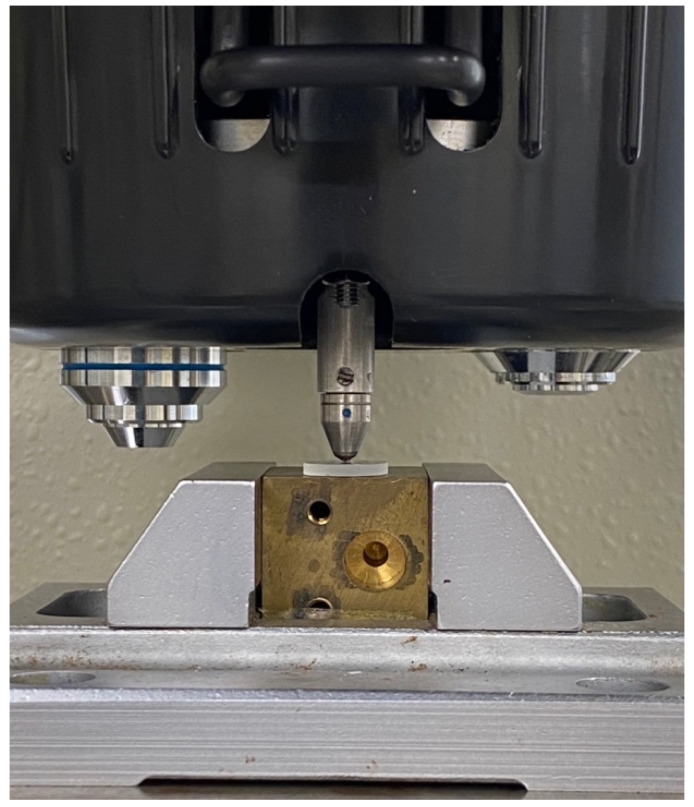
Vickers hardness test setup.

Disk-shaped specimens measuring 10 mm in diameter × 2 mm in thickness were used for the Vickers hardness test (n = 6). A Vickers hardness tester (MVK-H2; Akashi, Kanagawa, Japan) was used to measure the hardness under a load of 300 gf and a dwell time of 15 s (Figure 7). The final hardness value (VHN) of each specimen was calculated arithmetically by taking the average of the measurements obtained with five indentations.

### 4.7. SEM Analysis of Zirconia Filler and Specimen Fracture Surface

The particle size and distribution of the zirconia filler and the fracture surface of the specimens after the three-point bending tests were analyzed by scanning electron microscopy (SEM; JSM-7900F, JEOL, Tokyo, Japan). The specimens were attached to an aluminum stub and their surfaces were coated with a thin layer of platinum (E102 Ion Sputter, Hitachi, Tokyo, Japan). The samples were then analyzed at an acceleration voltage of 5 k, a discharge current of 8 μA, and a working distance of 6–10 mm. Microscopic images were acquired at a magnification of 40,000× (10 wt.%) for particle size, 3000× (0 (control), 3, 5, and 10 wt.%) for filler distribution, and 150× (5 and 10 wt.%) for the fracture surface of the specimens after the three-point bending tests. The average particle size was determined based on the SEM images using Fiji-ImageJ software (National Institutes of Health, Bethesda, MD, USA) with the linear intercept method. Particle size distribution was measured for a minimum of 600 particles.

### 4.8. Statistical Analysis

Tukey’s multiple comparison tests were carried out after one-way ANOVA for the average values of the flexural strength, flexural modulus, and Vickers hardness obtained (*p* < 0.05) using statistical analysis software (IBM SPSS statistics 22.0; IBM, Armonk, NY, USA).

## 5. Conclusions

Within the scope of this study, the following conclusions can be drawn:

1. Varying the zirconia filler content (0, 3, 10, and 5 wt.%) and printing directions (0° and 90°) affected the mechanical properties of the 3D-printed photopolymer resins.
2. When the printing direction was set to 0°, the optimum mechanical properties that met the ISO standard requirements were obtained at a zirconia content of 0 wt.% (control).
3. When the printing direction was set to 90°, the optimum mechanical properties that met the ISO standard requirements were obtained at a zirconia content of 3 wt.%.
4. Regardless of the printing direction (0° and 90°), adding 5 and 10 wt.% zirconia degraded the mechanical properties of the resins.
5. When the printing directions were set to 0° and 90°, the Vickers hardness increased in the order of 0 (control), 3, 5, and 10 wt.%.

## Figures and Tables

**Table 1:** Mean (MPa) and standard deviation (SD) values of the flexural strengths and moduli for the test groups in the 0° printing direction.

Printing Direction	Zirconia Filler Content (wt.%)	Flexural Strength (MPa)	Flexural Modulus (GPa)
0°	Control (0)	50.6 (0.8) ^a^	0.8 (0.03) ^d^
	3	48.4 (1.4) ^b^	0.7 (0.07) ^e^
	5	43.4 (0.8) ^c^	0.5 (0.05) ^f^
	10	43.5 (0.8) ^c^	0.5 (0.04) ^f^

Data are presented as mean (SD). One-way ANOVA among the four groups yielded *p* < 0.05. ^a–f^: Statistically significant differences are based on Tukey’s highly significant difference (HSD) test for the post hoc comparison test at *p* < 0.05. n = 6 (specimens per group).

**Table 2:** Mean (MPa) and SD values of the flexural strengths and moduli for the test groups in the 90° printing direction.

Printing Direction	Zirconia Filler Content (wt.%)	Flexural Strength (MPa)	Flexural Modulus (GPa)
90°	Control (0)	47.8 (1.1) ^a^	0.7 (0.04) ^d^
	3	51.0 (1.0) ^b^	0.9 (0.04) ^e^
	5	43.2 (0.5) ^c^	0.6 (0.03) ^f^
	10	43.7 (1.1) ^c^	0.7 (0.04) ^df^

Data are presented as mean (SD). One-way ANOVA among the four groups yielded *p* < 0.05. ^a–f^: Statistically significant differences are based on Tukey’s HSD test for the post hoc comparison test at *p* < 0.05. n = 6 (specimens per group).

**Table 3:** Mean (VHN) and standard deviation (SD) values of the Vickers hardness for the test groups in the 0° printing direction.

Printing Direction	Zirconia Filler Content (wt.%)	Vickers Hardness (VHN)
0°	Control (0)	15.7 (0.3) ^a^
	3	17.8 (0.2) ^b^
	5	19.3 (0.7) ^c^
	10	21.0 (0.4) ^d^

Data are presented as mean (SD). One-way ANOVA among the four groups yielded *p* < 0.05. ^a–d^: Statistically significant differences are based on Tukey’s highly significant difference (HSD) test for the post hoc comparison test at *p* < 0.05. n = 6 (specimens per group).

**Table 4:** Mean (VHN) and SD values of the Vickers hardness for the test groups in the 90° printing direction.

Printing Direction	Zirconia Filler Content (wt.%)	Vickers Hardness (VHN)
90°	Control (0)	15.9 (0.5) ^a^
	3	17.9 (0.2) ^b^
	5	20.8 (0.5) ^c^
	10	21.2 (0.4) ^c^

Data are presented as mean (SD). One-way ANOVA among the four groups yielded *p* < 0.05. ^a–c^: Statistically significant differences based on Tukey’s HSD test for the post hoc comparison test at *p* < 0.05. n = 6 (specimens per group).

**Table 5:** Details of the tested materials.

Material	Brand Name	Composition	Value (%)	Lot Number
Methacrylate-based photopolymer resin		Methacrylate oligomer	75–90	
	Clear V4	Methacrylate monomer	25–50	L-20210108c
	(RS-F2-GPCL-04)	Diphenyl (2,4,6-trimethylbenzoyl) phosphine oxide	<1	
**Material**	**Brand Name**	**Powder Properties**		
Highly translucent zirconia filler		Y_2_O_3_	4 mol%	
	Zpex4	Specific surface area	7 ± 2 m^2^/g	N/A
		Green density	3.2 g/cm^3^	
